# M6P/IGF2R modulates the invasiveness of liver cells via its capacity to bind mannose 6-phosphate residues

**DOI:** 10.1016/j.jhep.2012.03.026

**Published:** 2012-08

**Authors:** Verena Puxbaum, Elisabeth Nimmerfall, Christine Bäuerl, Nicole Taub, Pia-Maria Blaas, Johannes Wieser, Mario Mikula, Wolfgang Mikulits, Ken M. Ng, George C.T. Yeoh, Lukas Mach

**Affiliations:** 1 Department of Applied Genetics and Cell Biology, University of Natural Resources and Life Sciences, Muthgasse 18, A-1190 Vienna, Austria; 2 Department of Internal Medicine I, Institute of Cancer Research, Medical University of Vienna, Borschkegasse 8a, A-1090 Vienna, Austria; 3 School of Biomedical, Biomolecular and Chemical Sciences, The University of Western Australia, 35 Stirling Highway, M310, Crawley, WA 6009, Australia; 4 Laboratory for Cancer Medicine, Western Australian Institute for Medical Research, 50 Murray Street, Perth, WA 6000, Australia

**Keywords:** BSA, bovine serum albumin, ECM, extracellular matrix, FBS, fetal bovine serum, HCC, hepatocellular carcinoma, HGF, hepatocyte growth factor, IGF-II, insulin-like growth factor II, M6P, mannose 6-phosphate, M6P/IGF2R, mannose 6-phosphate/insulin-like growth factor II receptor, MPR46, 46-kDa mannose 6-phosphate receptor, RNAi, RNA interference, shRNA, short hairpin RNA, siRNA, short interfering RNA, Cathepsin, Hepatocellular carcinoma, Lysosome, Matrix degradation, Cell invasion

## Abstract

**Background & Aims:**

The mannose 6-phosphate/insulin-like growth factor II receptor (M6P/IGF2R), a multifunctional protein, plays a central role in intracellular targeting of lysosomal enzymes and control of insulin-like growth factor II (IGF-II) bioactivity. Importantly, the gene encoding this receptor is frequently inactivated in a wide range of malignant tumors including hepatocellular carcinomas. Thus, M6P/IGF2R is considered a putative liver tumor suppressor. The aim of this study was to establish the impact of the receptor on the invasive properties of liver cells.

**Methods:**

Reconstitution experiments were performed by expression of wild type and mutant M6P/IGF2R in receptor-deficient FRL14 fetal rat liver cells. RNA interference was used to induce M6P/IGF2R downregulation in receptor-positive MIM-1–4 mouse hepatocytes.

**Results:**

We show that the M6P/IGF2R status exerts a strong impact on the invasiveness of tumorigenic rodent liver cells. M6P/IGF2R-deficient fetal rat liver cells hypersecrete lysosomal cathepsins and penetrate extracellular matrix barriers in a cathepsin-dependent manner. Forced expression of M6P/IGF2R restores intracellular transport of cathepsins to lysosomes and concomitantly reduces the tumorigenicity and invasive potential of these cells. Conversely, M6P/IGF2R knock-down in receptor-positive mouse hepatocytes causes increased cathepsin secretion as well as enhanced cell motility and invasiveness. We also demonstrate that functional M6P-binding sites are important for the anti-invasive properties of M6P/IGF2R, whereas the capacity to bind IGF-II is dispensable for the anti-invasive activity of the receptor in liver cells.

**Conclusions:**

M6P/IGF2R restricts liver cell invasion by preventing the pericellular action of M6P-modified proteins.

## Introduction

Liver cancer represents worldwide one of the most frequent malignancies [1]. Hepatocellular carcinomas (HCCs) are the most common primary liver tumors. Although many improvements have been made in terms of diagnosis and treatment, HCCs are associated with poor clinical prognosis [2]. Aggressive HCCs have the capacity to penetrate extracellular matrix (ECM) barriers and spread into the surrounding parenchyma, leading to intrahepatic metastasis and portal venous invasion [3].

Different proteinases are involved in the breakdown of ECM components during tumor invasion and metastasis, including plasminogen activators, matrix metalloproteinases, and cathepsins [4–6]. Hepatocytes are known to produce substantial amounts of the lysosomal proteinases cathepsin B, cathepsin D, and cathepsin L [7]. As typical for lysosomal enzymes, the N-glycan moieties of cathepsins are modified during their biosynthesis with mannose 6-phosphate (M6P) residues which permit interaction with the main lysosomal sorting receptors, the 300-kDa mannose 6-phosphate/insulin-like growth factor II receptor (M6P/IGF2R) and the 46-kDa mannose 6-phosphate receptor (MPR46) [8].

M6P/IGF2R is a multifunctional receptor involved in (a) transport of newly synthesized M6P-tagged lysosomal proteins from the Golgi network to lysosomal compartments, (b) endocytosis of extracellular M6P-tagged lysosomal enzymes, (c) proteolytic activation of transforming growth factor β, and (d) regulation of the bioavailability of IGF-II [8–10]. To perform all these important functions, the receptor depends on multiple ligand-binding sites. For some M6P/IGF2R ligands, the receptor domains accommodating the respective binding sites have been identified. Furthermore, point mutations have been described which specifically interfere with the interaction of the receptor with IGF-II or M6P-tagged proteins [11–14].

Several lines of evidence support the hypothesis that loss of M6P/IGF2R function is associated with liver tumor progression. The gene encoding M6P/IGF2R has been shown to undergo frequent loss of heterozygosity in human HCCs and adenomas, with concomitant inactivating mutations in the remaining allele [15,16]. It has been demonstrated that the receptor gene is lost early in liver tumorigenesis, which suggests that loss of M6P/IGF2R may represent an initiation event [17]. Given this clinical significance, it comes as a surprise that the impact of the M6P/IGF2R status on the properties of hepatocytes and HCC cells has not yet been firmly established. However, studies in choriocarcinoma and breast cancer cells have demonstrated that a decrease in M6P/IGF2R expression enhances tumor cell growth [18,19], whereas overexpression of the receptor causes the opposite effect [20,21].

Experiments in transgenic mice have indicated that the anti-tumorigenic properties of M6P/IGF2R could be linked to its capacity to downregulate the biological activities of IGF-II [22]. However, other studies indicate that failure to express M6P/IGF2R may result in an increase of the proteolytic load in the pericellular environment and thus enhance the invasive capacity of tumor cells [23–25]. Thus, it seems that multiple M6P/IGF2R ligands play a role in tumor formation and metastasis, possibly in a tissue-specific manner. In this study, we have now assessed the impact of M6P/IGF2R on the growth, motility and invasiveness of liver cells.

## Materials and methods

A detailed account of the methodology used in this study can be found in the Supplementary Materials and methods.

## Results

### FRL14 cells display features typical for early-stage fetal hepatocytes

**Supplementary Figure 1:**
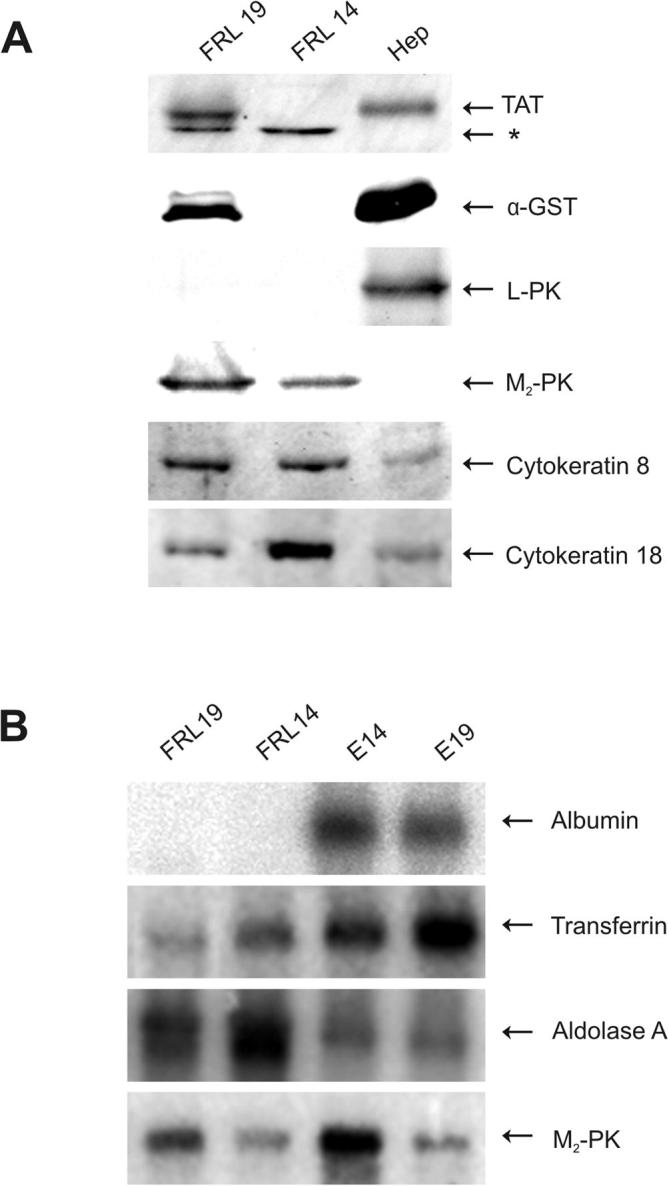
**Properties of parental FRL14 cells**. (A) Cell extracts (30 μg protein) were subjected to immunoblotting analysis using antibodies to tyrosine aminotransferase (TAT) and L-type pyruvate kinase (L-PK) [1]; alpha-type glutathione S-transferase (α-GST) [2]; M_2_-type pyruvate kinase (Rockland Immunochemicals, Gilbertsville, PA); cytokeratins 8 and 18 (DAKO, Carpinteria, CA). Hep, primary adult rat hepatocytes; ^∗^non-specific signal. (B) Northern blotting analysis of total RNA (30 μg) isolated from FRL14 and FRL19 cells as well as primary fetal rat hepatocytes (E14, E19) was performed as described [1,3].

There is now compelling evidence to suggest that in some liver pathologies HCCs derive from liver stem/progenitor cells or immature hepatoblasts. Hence, HCC cells often more closely resemble fetal than adult hepatocytes in terms of their gene expression pattern [26]. To permit detailed studies on a cellular level, substantial efforts have been undertaken to establish transformed hepatocyte-like cell lines from fetal rat liver of different gestational stages. One such cell line, FRL19, has been previously derived from a prenatal rat liver at day 18.5 of gestation and shown to express several hepatocyte markers, including the late-onset enzymes tyrosine aminotransferase and alpha-glutathione S-transferase [27,28]. We have now generated a cell line derived from day 13.5 fetal rat hepatocytes. The morphology of these FRL14 cells reflects their origin from the hepatocyte lineage. Furthermore, FRL14 cells express early-stage hepatocyte markers such as transferrin, while late-onset genes are not expressed (Supplementary Fig. 1).

### In vitro invasion of M6P/IGF2R-deficient FRL14 cells is strongly inhibited by cysteine cathepsin inhibitors

**Supplementary Figure 2:**
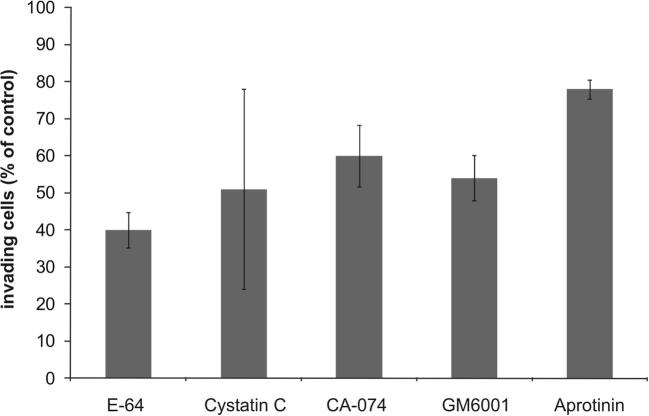
**Matrigel invasion of FRL14 cells in the presence of various proteinase inhibitors**. The final concentration was 1 μM (aprotinin, cystatin C) or 10 μM (CA-074, E-64, GM6001). An inactive derivative of the respective inhibitor (GM6001) or the solvent alone were used as controls. Data are presented as means ± SD of two independent experiments.

**Fig. 1:**
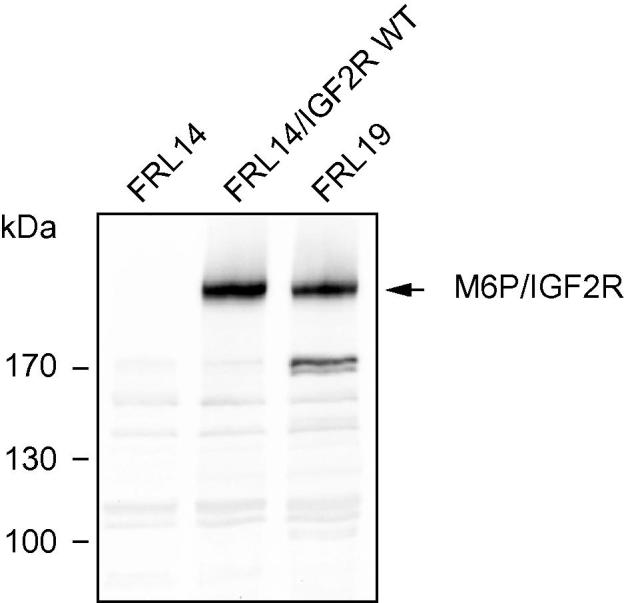
**M6P/IGF2R status of FRL14 cells**. Membrane protein extracts (40 μg) of FRL14, FRL14/IGF2R wt, and FRL19 cells were subjected to SDS–PAGE and then immunoblotted with anti-M6P/IGF2R antibodies. The migration positions of selected molecular mass standards are indicated.

FRL14 cells are capable of migrating across ECM barriers as typical for malignant cancer cells. Since such invasive properties frequently depend on ECM proteolysis, the contribution of matrix-degrading proteinases to the invasive potential of FRL14 cells was assessed by invasion assays performed in the presence of different synthetic and endogenous proteinase inhibitors (Supplementary Fig. 2). The strongest effect was caused by the general lysosomal cysteine proteinase inhibitor E-64 (60% reduction). Inhibition of FRL14 invasion by the more selective E-64 derivative CA-074 (40%) and the matrix-metalloproteinase inhibitor GM6001 (46%) was less pronounced. When the efficacy of physiological proteinase inhibitors was tested, it was found that the potent serine proteinase inhibitor aprotinin had a much weaker effect on cell invasion (22% reduction) than the cysteine proteinase inhibitor cystatin C (49% reduction). We have earlier observed a similar proteinase inhibition profile for the invasive properties of M6P/IGF2R-deficient murine squamous cell carcinoma cells [25]. When FRL14 extracts were immunoblotted with antibodies recognizing rat M6P/IGF2R, it became evident that these cells lack expression of the receptor (Fig. 1).

### Ectopic expression of M6P/IGF2R restores the intracellular retention of lysosomal enzymes and decreases the invasive potential of FRL14 cells in vitro

**Supplementary Figure 3:**
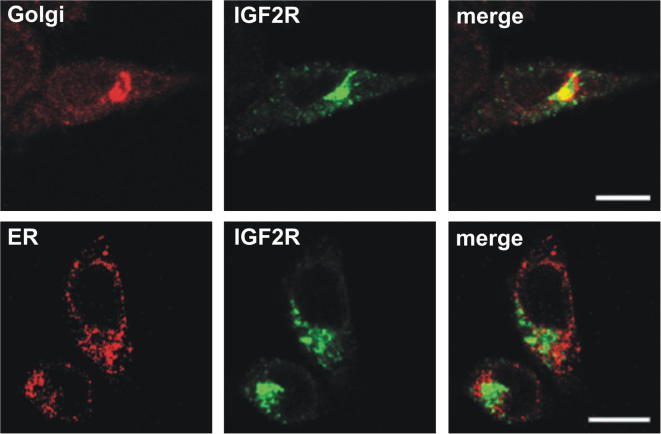
**Confocal immunofluorescence images of FRL14 cells expressing wild-type M6P/IGF2R**. Cells were double-stained with antibodies against M6P/IGF2R and Golgi-58K protein or protein disulfide isomerase (endoplasmic reticulum). Scale bars, 10 μm.

Receptor-deficient FRL14 cells were stably transfected with human wild type *M6P/IGF2R* cDNA to assess the impact of the M6P/IGF2R status on their cellular properties. Two clones were selected for further studies, FRL14/IGF2R wt-1 and FRL14/IGF2R wt-2. By comparison with receptor-positive HeLa human cervical carcinoma cells, the M6P/IGF2R content of FRL14/IGF2R wt-1 and FRL14/IGF2R wt-2 cells was estimated to be 2.5 and 2.1 pmol/mg total cell protein, respectively (Supplementary Table 1). Hence, the receptor level of the selected clones was within the physiological range [29]. The subcellular localization of M6P/IGF2R was assessed by immunofluorescence microscopy. As expected, the ectopically expressed receptor was found to reside in the Golgi apparatus (Supplementary Fig. 3).

**Supplementary Figure 4:**
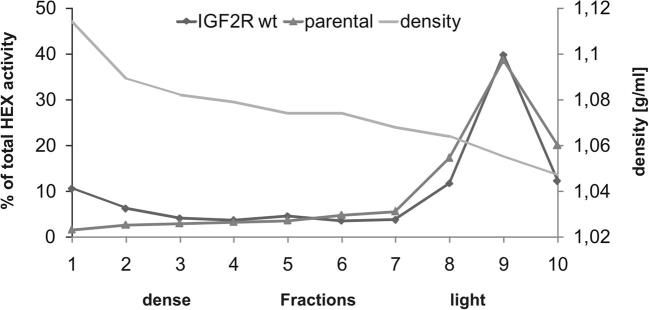
**Subcellular fractionation of parental and M6P/IGF2R-expressing FRL14 cells**. Post-nuclear supernatants were subjected to subcellular fractionation in 25.7% Percoll gradients. The fractions thus obtained were then analyzed for their buoyant density and β-*N*-acetylhexosaminidase (HEX) activity. Marker enzymes for the endoplasmic reticulum (NADH-cytochrome c reductase) and the Golgi apparatus (galactosyltransferase) were only detected in the light gradient fractions.

**Fig. 2:**
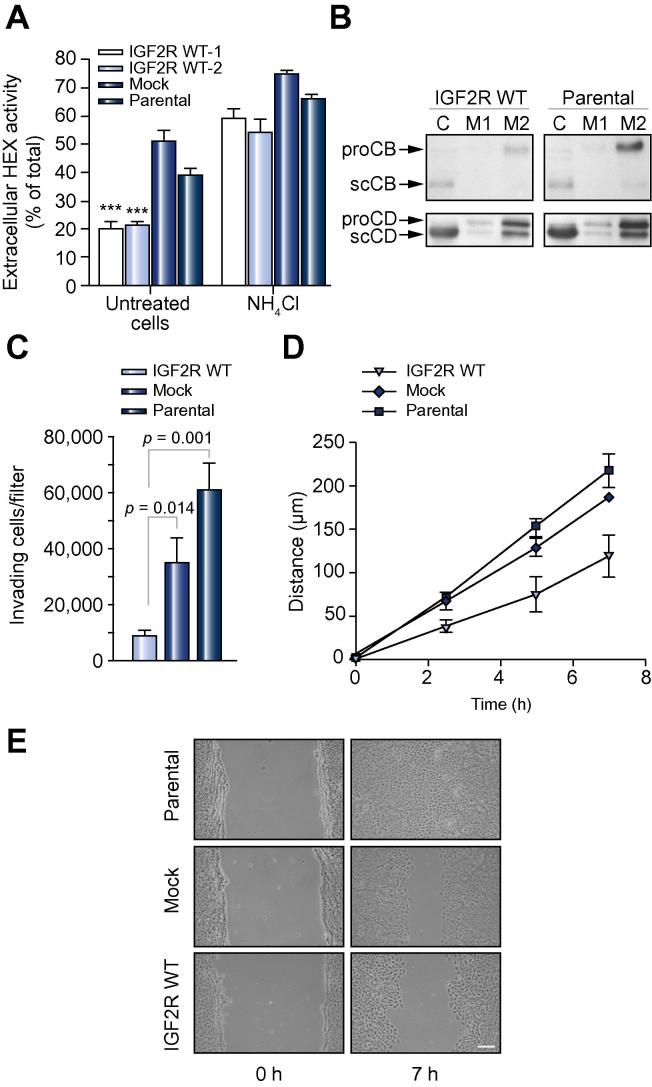
**M6P/IGF2R reduces lysosomal enzyme secretion and Matrigel invasion by FRL14 cells**. (A) Two clones of M6P/IGF2R-expressing FRL14 cells (IGF2R wt-1 and IGF2R wt-2) as well as parental and mock-transfected cells were incubated for 24 h with or without 10 mM NH_4_Cl prior to determination of β-*N*-acetylhexosaminidase (HEX) activity in cell lysates and media. The secretion levels are presented as means ± SEM of 4–9 independent experiments. ^∗∗∗^*p* <0.001 compared to mock-transfected cells. (B) Microsomal extracts of IGF2R wt-1 and parental cells (C; 20 μg total protein) and medium corresponding to 20 μg (M1) and 100 μg (M2) total microsomal protein were subjected to SDS–PAGE and then immunoblotted with antibodies to cathepsin B (CB) and cathepsin D (CD). proCB/proCD, procathepsin B/D; scCB/scCD, mature single-chain cathepsin B/D. (C) Invasion assays of IGF2R wt-1, parental and mock-transfected FRL14 cells using 10% FBS as chemoattractant. Data are presented as means ± SEM of 4 independent experiments. (D) Wound healing assays of IGF2R wt-2, parental and mock-transfected FRL14 cells. Data are presented as means ± SD of 3 wounds. Some error bars have been omitted to avoid obstruction of individual data points. *p* = 0.006 compared to parental cells; *p* = 0.04 compared to mock-transfected cells. (E) Phase contrast microscopy images of representative wounds at the time of wounding (0 h) and after incubation for 7 h. Scale bar, 100 μm.

M6P/IGF2R transports newly synthesized lysosomal enzymes from the Golgi network to lysosomal compartments [30,31]. To test the sorting function of the recombinant receptor, we analyzed the intra- and extracellular activity of the lysosomal marker β-*N*-acetylhexosaminidase. Due to the lack of M6P/IGF2R, parental and mock-transfected FRL14 cells secrete large amounts of β-*N*-acetylhexosaminidase (39 ± 2% and 51 ± 3%, respectively). Ectopic expression of human M6P/IGF2R reduced β-*N*-acetylhexosaminidase secretion considerably (FRL14/IGF2R wt-1: 21 ± 2%, FRL14/IGF2R wt-2: 21 ± 1%). The addition of NH_4_Cl, a lysosomotropic base interfering with M6P/IGF2R function [23,32], strongly increased secretion of β-*N*-acetylhexosaminidase by FRL14/IGF2R wt cells. The fact that also both parental and mock-transfected FRL14 cells showed an increase in β-*N*-acetylhexosaminidase secretion upon NH_4_Cl treatment is probably due to the presence of MPR46, which also transports lysosomal enzymes in an NH_4_Cl-sensitive manner (Fig. 2A). Additionally, the intracellular transport of cathepsin B and cathepsin D was analyzed. Substantially more procathepsin B was found in the medium of parental FRL14 cells (38% secretion) as compared to M6P/IGF2R transfectants (16% secretion). For cathepsin D, the difference in secretion between FRL14/IGF2R wt (21% secretion) and parental FRL14 cells (27% secretion) was not as pronounced (Fig. 2B). When FRL14/IGF2R wt cells were subjected to subcellular fractionation by Percoll density-gradient centrifugation, 21% of the total β-*N*-acetylhexosaminidase activity was found in the dense (lysosomal) fraction, whereas in parental FRL14 cells only 7% of this enzyme resides in lysosomes (Supplementary Fig. 4). These results demonstrate that ectopic expression of M6P/IGF2R in FRL14 cells leads to improved intracellular retention of lysosomal enzymes and restoration of dense lysosome formation, as previously shown for receptor-deficient squamous cell carcinoma cells [25].

It has been established that the metastatic properties of tumor cell lines closely correlate with the ability of the cells to migrate across Matrigel barriers *in vitro* [33]. The invasiveness of M6P/IGF2R-expressing FRL14 cells was found to be reduced by 76% compared to mock-transfected FRL14 cells, and by 86% compared to parental FRL14 cells (Fig. 2C). This indicates that M6P/IGF2R is indeed a potent anti-invasive factor in liver cells.

The motility of parental and receptor-expressing FRL14 cells was also tested in wound healing assays. Parental FRL14 monolayers were highly active in wound closure. After 7 h, all wounds were fully closed, reflecting a mean covered distance of 218 μm. Mock-transfected FRL14 cells behaved similar to parental cells (186 μm). In contrast, the wounds of FRL14/IGF2R wt cells (118 μm) were only about half-closed after this incubation period. These results show that the presence of M6P/IGF2R strongly reduces the motility of FRL14 cells *in vitro* (Fig. 2D and E).

### Anchorage-independent proliferation and tumor growth are reduced by reconstitution of M6P/IGF2R expression in FRL14 cells

Anchorage-independent growth is believed to be a hallmark of cellular transformation [34]. Soft-agar assays were performed to test whether FRL14 cells are able to grow in an anchorage-independent manner. The colony-formation efficiency of FRL14/IGF2R wt cells (81 ± 6 colonies) was considerably lower than that of parental FRL14 cells (466 ± 23 colonies; *p* <0.001). In addition, colonies of FRL14/IGF2R wt cells (0.013 ± 0.002 mm^2^) were much smaller than those of FRL14 parental cells (0.037 ± 0.005 mm^2^; *p* = 0.005).

Furthermore, we tested the ability of FRL14 cells to form tumors in immunodeficient mice. Contrary to parental FRL14 cells (tumors/injections: 9/9; tumor weight: 13–219 mg; median: 48 mg), tumor formation by FRL14/IGF2R wt cells was reduced and the tumors were smaller (tumors/injections: 5/9; tumor weight: 0–100 mg; median: 18 mg). Collectively, our results thus indicate that reconstitution of functional M6P/IGF2R expression interferes with FRL14 tumor progression.

### The relevance of individual ligand-binding sites for the anti-invasive activity of M6P/IGF2R

M6P/IGF2R is a multifunctional protein with separate binding sites for IGF-II (domain 11) and M6P-modified ligands (domains 3 and 9). These binding sites were individually mutated, thus creating M6P/IGF2R variants either impaired in their interaction with IGF-II (M6P/IGF2R dom11^mut^) or M6P (M6P/IGF2R dom3/9^mut^). Parental FRL14 cells were then stably transfected with M6P/IGF2R dom11^mut^ and M6P/IGF2R dom3/9^mut^ cDNAs. The receptor content of the resulting cell lines was found to be similar to or slightly above that of FRL14/IGF2R wt cells (Supplementary Table 1). Indirect immunofluorescence studies revealed that the subcellular distribution of M6P/IGF2R dom11^mut^ and M6P/IGF2R dom3/9^mut^ is identical to that of the wild type receptor. This confirms that these mutations do not compromise folding and/or intracellular targeting of M6P/IGF2R in FRL14 cells.

**Supplementary Figure 5:**
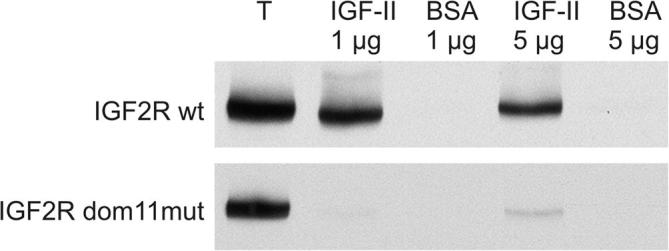
**Characterization of M6P/IGF2R dom11^mut^ expressed in FRL14 cells**. Membrane extracts of FRL14 cells expressing either wild-type or mutant M6P/IGF2R were incubated with different amounts of biotinylated IGF-II or BSA prior to addition of avidin-Sepharose beads. Bound material was then subjected to immunoblotting analysis with anti-M6P/IGF2R antibodies. T, total material applied to the beads.

**Fig. 3:**
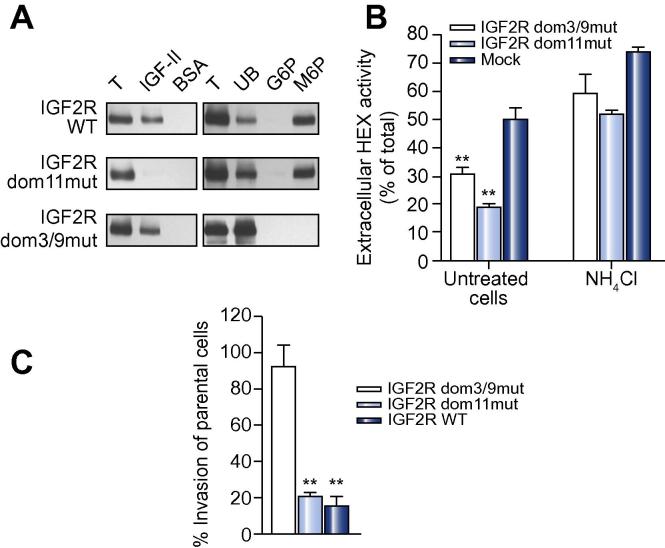
**Characterization of M6P/IGF2R mutants expressed in FRL14 cells**. (A) Left panel: membrane extracts of FRL14 cells expressing either wild type or mutant M6P/IGF2R were incubated with 1 μg biotinylated IGF-II or BSA prior to addition of avidin–Sepharose beads. Bound material was then subjected to immunoblotting analysis with anti-M6P/IGF2R antibodies. T, total material applied to the beads. Right panel: membrane extracts of FRL14 cells expressing either wild type or mutant M6P/IGF2R were incubated with phosphomannan–Sepharose beads. After washing with glucose 6-phosphate (G6P), bound proteins were eluted with 5 mM M6P prior to immunoblotting analysis as above. UB, unbound material. These experiments were repeated 2–5 times with similar results. (B) Cells were incubated for 24 h with or without 10 mM NH_4_Cl prior to determination of β-*N*-acetylhexosaminidase (HEX) activity in cell lysates and media. The secretion levels are presented as means ± SEM of 3–9 independent experiments. ^∗∗^*p* <0.01 compared to mock-transfected cells. See Fig. 2A for the results obtained with cells expressing wild type M6P/IGF2R. (C) Invasion assays of FRL14 cells expressing either wild type or mutant M6P/IGF2R using 10% FBS as chemoattractant. Data are presented as means ± SEM of 4 independent experiments. ^∗∗^*p* <0.01 compared to parental cells.

Wild type and mutant forms of M6P/IGF2R were subjected to IGF-II and phosphomannan binding assays. As expected, the wild type receptor was able to bind both ligands. The interaction of M6P/IGF2R dom11^mut^ with IGF-II was much weaker, while this mutation did not impede binding of M6P. The M6P binding site mutant M6P/IGF2R dom3/9^mut^ showed no detectable binding of M6P, but the ability to interact with IGF-II was preserved (Fig. 3A and Supplementary Fig. 5).

To study the function of the mutant receptors, the intra- and extracellular activity of β-*N*-acetylhexosaminidase was measured. FRL14/IGF2R dom11^mut^ cells secreted 19 ± 1% of their β-*N*-acetylhexosaminidase activity, which is similar to cells expressing the wild type receptor (21% secretion). Both FRL14/IGF2R dom3/9^mut^ clones secreted a larger proportion of this enzyme (31 ± 1% in either case), but β-*N*-acetylhexosaminidase secretion is not as pronounced as in mock-transfected cells (51%). The addition of NH_4_Cl strongly increased the secretion of β-*N*-acetylhexosaminidase by all cell lines tested, including those expressing mutant receptors (Fig. 3B).

As already observed for FRL14/IGF2R wt cells, the invasive potential of FRL14/IGF2R dom11^mut^ cells was found to be strongly diminished compared to parental FRL14 cells (80% reduction). In contrast, the invasive capacity of FRL14/IGF2R dom3/9^mut^ cells (14% reduction) was only slightly lower than that of parental FRL14 cells. This suggests that the ability to bind IGF-II is dispensable for the anti-invasive activity of M6P/IGF2R in liver cells. In contrast, the anti-invasive potential of the receptor appears to be largely based on its ability to interact with M6P-modified ligands (Fig. 3C).

### RNAi-mediated knock-down of M6P/IGF2R increases secretion of lysosomal enzymes and in vitro invasion of MIM-1–4 cells

**Supplementary Figure 6:**
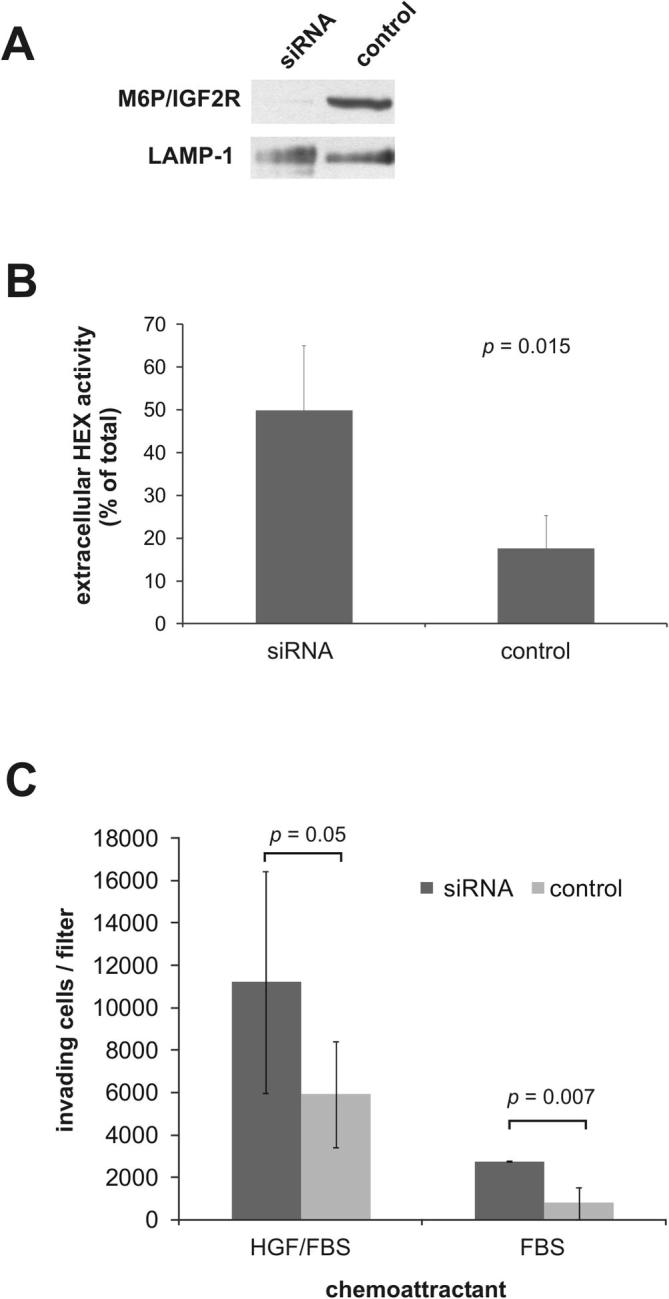
**Effects of transient M6P/IGF2R knock-down on MIM-1-4 cells**. (A) Transfection of MIM-1–4 cells with siRNA oligonucleotides or transfection reagent alone (control). Membrane extracts (50 μg protein) were then immunoblotted with anti-M6P/IGF2R antibodies. LAMP-1 was used as loading control. (B) Transfected cells were incubated for 24 h prior to determination of β-*N*-acetylhexosaminidase (HEX) activity in cell lysates and media. The secretion levels are presented as means ± SD of 4 independent experiments. (C) Invasion assays using 10% FBS ± 10 ng/ml mouse HGF as chemoattractant. Data are presented as means ± SEM of 3–6 independent experiments.

RNAi-mediated gene silencing was used to knock-down M6P/IGF2R expression in receptor-positive MIM-1–4 mouse hepatocytes. For transient knock-down studies, MIM-1–4 cells were transfected with siRNA oligonucleotides targeting *Igf2r* mRNA. Densitometric analysis of immunoblots revealed that siRNA treatment reduced the receptor content of the cells to <5%. This almost quantitative depletion of endogenous M6P/IGF2R provoked a strong increase in the secretion of β-*N*-acetylhexosaminidase (37 ± 1%) as compared to mock-transfected cells (11 ± 1%). The invasive potential of siRNA-treated MIM-1–4 cells was also markedly higher than that of the respective controls, using either HGF/FBS (1.9-fold increase) or FBS alone (3.8-fold increase) as chemoattractant (Supplementary Fig. 6).

**Fig. 4:**
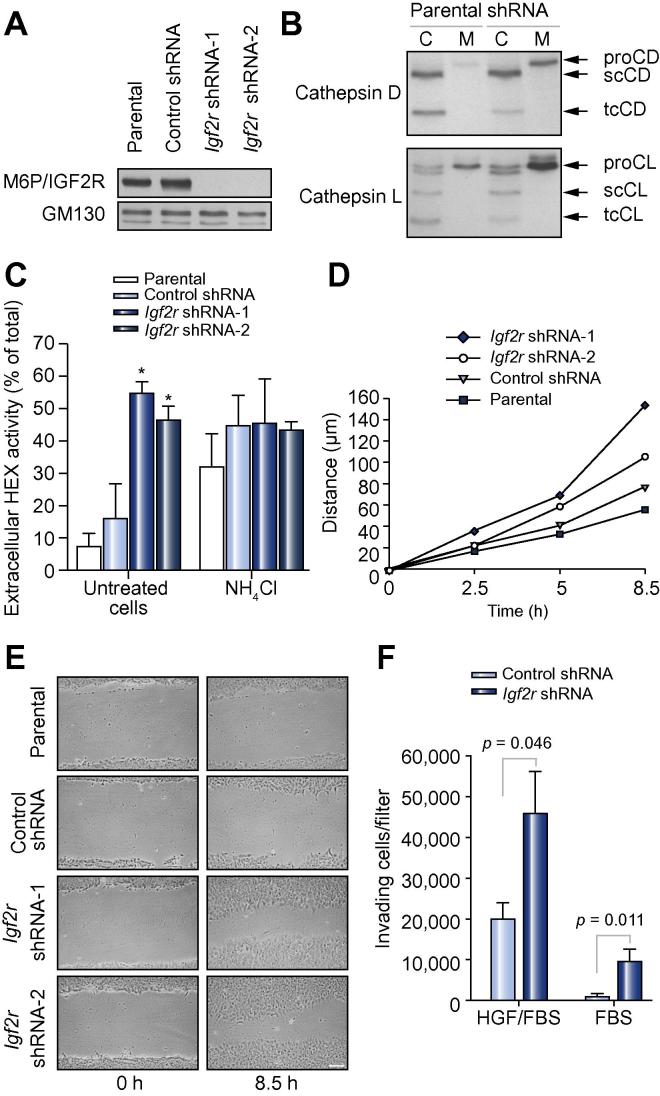
**Effects of stable M6P/IGF2R knock-down on MIM-1–4 cells**. (A) Immunoblot of membrane extracts with antibodies against M6P/IGF2R. GM130 was used as loading control. (B) Cell lysates (C; 20 μg protein) and conditioned media (M) corresponding to 100 μg (CD) or 4 μg (CL) total cellular protein were subjected to immunoblotting with antibodies to cathepsin D (CD) or cathepsin L (CL). proCD/proCL, procathepsin D/L; scCD/scCL, mature single-chain cathepsin D/L; tcCD/tcCL, mature two-chain cathepsin D/L. (C) Cells were incubated for 24 h with or without 10 mM NH_4_Cl prior to determination of β-*N*-acetylhexosaminidase (HEX) activity in cell lysates and media. The secretion levels are presented as means ± SEM of three independent experiments. ^∗^*p *< 0.05 compared to cells transfected with control shRNA. (D) Analysis of cell migration using the monolayer wound healing assay. Data are presented as means of 3–4 wounds. Error bars have been omitted to avoid obstruction of the individual data points. *IGF2R* shRNA-1: *p* = 0.001 compared to parental cells; *p* = 0.044 compared to cells transfected with control shRNA. (E) Phase contrast microscopy images of representative wounds at the time of wounding (0 h) and after incubation for 8.5 h. Scale bar, 100 μm. (F) Invasion assays using 10% FBS ± 10 ng/ml mouse HGF as chemoattractant. Data are presented as means ± SEM of five independent experiments.

For permanent receptor knock-down, MIM-1–4 cells were stably transfected with an *M6P/IGF2R*-specific shRNA construct. Densitometric analysis of immunoblots revealed that the residual receptor content of the two *MIM-1–4/IGF2R* shRNA clones chosen for further studies was <1% as compared with cells transfected with a control shRNA sequence (Fig. 4A). This goes in hand with enhanced β-*N*-acetylhexosaminidase secretion, with *MIM-1–4/IGF2R* shRNA cells (line 1: 55%; line 2: 47%) secreting far more of this lysosomal marker enzyme than parental (7%) and control cells (16%). Similar observations were made for cathepsin D. *MIM-1–4/IGF2R* shRNA cells secreted substantially more of this lysosomal proteinase (11%) than parental MIM**-**1–4 cells (1%). The difference in cathepsin L secretion between *MIM-1–4/IGF2R* shRNA and parental cells was less pronounced (87% and 81%, respectively). NH_4_Cl treatment resulted in strongly enhanced β-*N*-acetylhexosaminidase secretion by parental and control MIM-1–4 cells. Conversely, *MIM-1–4/IGF2R* shRNA cells displayed no further increase in β-*N*-acetylhexosaminidase secretion upon addition of NH_4_Cl (Fig. 4B and C).

Wound healing assays were performed to test the effect of M6P/IGF2R knock-down on the motility of MIM-1–4 cells. *MIM-1–4/IGF2R* shRNA-1 (mean covered distance: 152 μm) and *MIM-1–4/IGF2R* shRNA-2 cells (106 μm) migrated substantially faster than parental (56 μm) and control cells (78 μm; Fig. 4D and E). In Matrigel invasion assays performed with HGF/FBS as chemoattractant, *MIM-1–4/IGF2R* shRNA-1 cells proved 2.3-fold more invasive than control cells. *MIM-1–4/IGF2R* shRNA-1 cells also displayed considerable invasiveness in the absence of exogenous HGF, while FBS alone was not sufficient to induce an appreciable invasive response by control cells (Fig. 4F).

## Discussion

The gene encoding M6P/IGF2R is frequently mutated during human and rodent hepatocarcinogenesis [35,36], and some of these mutations have been shown to inactivate individual receptor functions [12,13]. However, the role of aberrant M6P/IGF2R expression in HCC formation and progression is still unknown. In this study, we have assessed the impact of the M6P/IGF2R status on the tumorigenic and invasive properties of a receptor-negative transformed fetal rat liver cell line (FRL14). We have found that reconstitution of M6P/IGF2R expression in FRL14 cells suppresses their tumorigenicity and invasiveness. Furthermore, the reconstituted cells were less motile and displayed diminished growth under anchorage-independent conditions. *Vice versa*, RNAi-mediated M6P/IGF2R knock-down in receptor-positive mouse hepatocytes enhanced their migratory and invasive potential. These results clearly indicate a mechanistic link between dysfunctional M6P/IGF2R expression and HCC pathogenesis, in agreement with the tumor-suppressive activities of the receptor in other forms of cancer [22,37].

In squamous cell carcinoma cells, the anti-invasive activity of M6P/IGF2R appears to rely on restriction of the pericellular accumulation of M6P-tagged lysosomal proteinases [23–25]. In contrast, the anti-invasive effects of M6P/IGF2R in breast cancer cells have been attributed to decreased IGF-II bioavailability [21]. Moreover, evidence has been provided that the anti-invasive properties of M6P/IGF2R in renal carcinoma cells are based on downregulation of cell-mediated plasminogen activation [38]. In our further studies, we therefore sought to establish the importance of the individual receptor-ligand interactions for the anti-invasive activity of M6P/IGF2R in liver cells. We selected to focus on the capacity of M6P/IGF2R to bind IGF-II and M6P-containing ligands, since plasmin was found to play only a minor role in FRL14 cell invasion. Furthermore, IGF-II has been recently found to enhance the motility of human HCC cells [39]. Structure-function studies have led to the identification of the M6P/IGF2R domains accounting for binding of IGF-II (domain 11) and M6P residues (domains 3 and 9). Point mutations within these domains have been described that selectively interfere with either interaction [11,14]. The introduction of a mutation known to reduce the affinity of M6P/IGF2R for IGF-II did not impede its anti-invasive capacity in FRL14 cells. Conversely, simultaneous mutation of both M6P-binding sites almost abolished this activity of the receptor. These results strongly support the notion that the anti-invasive properties of M6P/IGF2R in liver cells depend on its interaction with M6P-tagged lysosomal hydrolase(s). Various lysosomal enzymes have been implicated to participate in the process of tumor invasion and metastasis. So far, the lysosomal enzymes most extensively studied in this context are cysteine cathepsins [6].

Liver tumor cells are known to secrete large amounts of cathepsins into the surrounding tissue [40]. Therefore, it can be envisaged that absence of M6P/IGF2R fosters HCC invasion by exacerbating the secretion of these potent proteinases. Indeed, we have observed that treatment with cysteine cathepsin inhibitors results in substantial reduction of the invasiveness of FRL14 cells. The main cellular targets of these inhibitors in liver cells are the two most prominent cysteine cathepsins, cathepsin B, and cathepsin L [7]. For cathepsin B, it has been shown previously that this enzyme may exhibit extracellular functions under pathophysiological conditions [41]. Compelling evidence for a major role of cathepsin B and related proteinases in tumor invasion and metastasis has been derived from pancreatic and breast cancer models in cathepsin knock-out mice [42–44]. Furthermore, transgenic mice overexpressing human cathepsin B have been found to develop breast cancer metastasis at a much higher rate than their control littermates [45]. Intriguingly, treatment of rodents with a broad-spectrum cysteine cathepsin inhibitor has proven effective in halting breast cancer progression when combined with other chemotherapeutic agents, encouraging consideration of cysteine cathepsins as therapeutic targets in human cancers [46]. However, it remains to be established whether pharmacological intervention with tumor-associated cysteine cathepsin activity holds promise for HCC treatment.

## Conflict of interest

The authors who have taken part in this study declare that they do not have anything to disclose regarding conflict of interest with respect to this manuscript.

## Financial support

Austrian Science Fund (FWF): P16925-B11; Austrian Science Fund (FWF): P20918-B11; Austrian Academy of Sciences: H-7/2005.

## Appendix A Supplementary data

Supplementary data Supplementary Materials and methods

Supplementary Table 1 M6P/IGF2R content of FRL14 cells expressing human M6P/IGF2R.

Supplementary Table 2 PCR primer sets for generation of mutant M6P/IGF2R cDNAs.
