# Supplementary material for: M6P/IGF2R modulates the invasiveness of liver cells via its capacity to bind mannose 6-phosphate residues
Source: J Hepatol. 2012 Aug;57(2):337–43. doi: 10.1016/j.jhep.2012.03.026 (PMC3401376; doi:10.1016/j.jhep.2012.03.026)
Supplement: Supplementary data — Supplementary Materials and methods [file mmc1.doc]

**Supplementary Materials and methods**

*Cell culture*

FRL14 and FRL19 [1] cells were propagated in Dulbecco’s Modified Eagle’s medium (DMEM) supplemented with 10% fetal bovine serum (FBS), 2 mM glutamine, 100 units/ml penicillin, 100 µg/ml streptomycin, 10^-8^ M dexamethasone (Sigma, St. Louis, MO) and 10^-8^ M insulin (Sigma). The FRL14 cell line was established essentially as reported previously [1], following transfection of primary fetal rat hepatocytes (developmental stage: day 13.5; [4]) with a plasmid containing SV40 T-antigen cDNA driven by the hepatocyte-selective retinol-binding protein promoter [5]. HeLa human cervical carcinoma cells (from the American Type Culture Collection, Manassas, VA) were cultured in DMEM supplemented with 10% FBS, 2 mM glutamine, 100 units/ml penicillin and 100 µg/ml streptomycin. Immortalized MIM-1-4 p19^ARF^ null hepatocytes [6] were cultured in RPMI 1640 supplemented with 10% FBS, 100 units/ml penicillin, 100 µg/ml streptomycin, 40 ng/ml transforming growth factor-α (Sigma), 30 ng/ml IGF-II (Sigma) and 1.4 nM insulin (Sigma), using collagen-coated plates.

*Heterologous expression of wild-type and mutant human M6P/IGF2R cDNAs in FRL14 cells*

Wild-type human M6P/IGF2R cDNA was cloned into the pAHygCMV2 expression vector as previously described [7]. Different strategies were used for the generation of mutant forms of M6P/IGF2R. The M6P/IGF2R dom11^mut^ point mutation (Ile^1572^→Thr) was generated with the Quick-change site-directed mutagenesis kit (Stratagene, La Jolla, CA) using *Pfu* DNA polymerase (Stratagene), a plasmid containing nucleotides 4469-5610 of the human M6P/IGF2R open reading frame as template and a complementary primer pair (nucleotides 4848-4876 with a T→C substitution at position 4862). The mutated sequence (nucleotides 4526-5521) was then excised with *PshA*I and inserted into wild-type M6P/IGF2R cDNA at the corresponding site. The resulting construct was then cloned into pAHygCMV2. The M6P/IGF2R dom3^mut^ (Arg^426^→Lys) and M6P/IGF2R dom9^mut^ (Arg^1325^→Lys) fragments were generated by means of overlap extension PCR [8] (see Supplementary Tab. 2 for primers and target sequences used) and then digested with *Msc*I/*Nsi*I (M6P/IGF2R dom3^mut^) or *Mph1103*I/*Stu*I (M6P/IGF2R dom9^mut^) prior to ligation with wild-type M6P/IGF2R cDNA cleaved with the same enzymes. The resulting mutant M6P/IGF2R sequences were then transferred into pAHygCMV2 as above. For production of the M6P/IGF2R dom3/9^mut^ construct, the fragment released by digestion of M6P/IGF2R dom3^mut^ cDNA with *Mph1103*I and *Mls*I was inserted into M6P/IGF2R dom9^mut^ cDNA cleaved with the same enzymes.

FRL14 cells were transfected with the different M6P/IGF2R constructs using Lipofectin (Invitrogen, Carlsbad, CA) according to the manufacturer’s instructions. Mock-transfected cells were generated by transfection with the parental vector pAHygCMV2. Selection of stably transfected cells was achieved by virtue of their ability to grow in the presence of 300 µg/ml hygromycin B (Invitrogen). Drug-resistant colonies were isolated after 14 days and tested for their M6P/IGF2R content by immunofluorescence staining and immunoblotting. Suitable clones were then expanded for further studies.

*RNA interference*

For transient M6P/IGF2R knock-down, small interfering RNA (siRNA) oligonucleotides targeting mouse M6P/IGF2R mRNA (target sequence: 5’-GAGCUAUGAUGAAUGUGUA-3’) were obtained from Invitrogen. MIM-1-4 cells were transfected with pre-annealed oligonucleotides using Dharma*FECT* 4 (Dharmacon, Lafayette, CO) according to the manufacturer’s instructions. Control cultures were treated using the transfection reagent only. Experiments were performed 72 hours after transfection.

Stable knock-down of M6P/IGF2R was achieved by transfection with a short hairpin RNA (shRNA) expression cassette. For this, a mouse M6P/IGF2R shRNA sequence (5’-gatcccc
GAGCTATGATGAATGTGTttcaagagaTACACATTCATCATAGCTCttttta-3’) was cloned into the pSUPER vector (OligoEngine, Seattle, WA) according to the manufacturer’s instructions. A rat M6P/IGF2R shRNA sequence (5’-gatccccGGGGAGACCTATGACGAAT
ttcaagagaATTCGTCATAGGTCTCCCCttttta-3’) was used as a control.

MIM-1-4 cells were transfected with these vectors using Lipofectin (Invitrogen) according to the manufacturer’s instructions. Selection of stably transfected cells was achieved by virtue of their ability to grow in the presence of 150 µg/ml hygromycin B (Invitrogen). Drug-resistant colonies were isolated after 14 days and tested for knock-down efficiency. Suitable clones were then expanded for further studies.

*Preparation of total membrane protein extracts*

Confluent cell monolayers (~10^7^ cells) were harvested in 500 µl PBS containing proteinase inhibitors (1 mM PMSF, 5 µg/ml E-64, 5 µg/ml leupeptin) and disrupted by ultrasonication. Post-nuclear supernatants were obtained by low-speed centrifugation (5 min at 320 × g, followed by 5 min at 800 × g) and then centrifuged for 60 min at 105000 × g. The pelleted membranes were either dissolved in 100 µl PBS containing 1% (w/v) SDS and proteinase inhibitors (immunoblotting) or extracted as described below (binding assays).

*Phosphomannan binding assays*

Total cellular membranes were resuspended in 100 µl binding buffer (0.15 M NaCl, 50 mM imidazole/HCl pH 7.0, 0.02% NaN_3_) containing proteinase inhibitors and extracted with 1% (w/v) Triton X-100 for 30 min at 0°C. Aliquots of these membrane extracts corresponding to 150 µg total protein were diluted ten-fold with binding buffer and incubated with 40 µl settled phosphomannan-Sepharose beads [9] on an end-over-end mixer for 16 h at 4°C. The beads were then washed 5 times with 1 ml binding buffer containing 0.1% Triton X-100. After another wash with 40 µl 5 mM glucose 6-phosphate (Sigma), specifically bound material was eluted with 40 µl 5 mM M6P (Sigma) in binding buffer.

*IGF-II binding assays*

Total cellular membrane extracts (100 µg of total protein) were diluted ten-fold with binding buffer (0.4 M KCl, 50 mM imidazole/HCl pH 7.0, 0.02% NaN_3_ containing 0.1% Triton X-100). The samples were then incubated either with 1 µg biotinylated IGF-II (Gropep, Adelaide, Australia) or BSA [9] for 16 h at 4°C on an end-over-end mixer, prior to capture of the biotinylated proteins with 40 µl avidin-Sepharose beads [9] for another 16 h at 4°C. The beads were then washed 5 times with 1 ml binding buffer and twice with 1 ml 10 mM Tris/HCl, pH 6.8. Finally, bound proteins were eluted with SDS-PAGE sample buffer by incubation for 5 min at 65°C.

*Wound healing assays*

Cells were seeded into 6-well culture dishes and grown to confluence. At least three parallel scratches were made into the confluent cell layer, using a 200-µl pipet tip. Afterwards, the cell layers were incubated in serum-free medium for up to 9 hours. Wound closure was followed using an inverted microscope equipped with a digital camera. Images of the wounds (at least 3 different areas per wound) were taken at the start and then at regular intervals. Finally, the images were analyzed using Adobe Photoshop 7.0. The cell migration distance was determined by measuring the half-widths of the wounds [10].

*Tumor formation assays*

*In vivo* tumor formation assays were performed using female severe combined immunodeficient mice (CB-17 SCID; Harlan Winckelmann, Borchen, Germany) aged 6-10 weeks. Cells were grown to confluence, trypsinized and resuspended each as single cell suspension in sterile Ringer’s solution. Each mouse received a single subcutaneous injection of 3 × 10^6^ cells in 100 µl Ringer’s solution into one of its rear flanks. The mice were sacrificed 6 weeks after inoculation, and the tumors were excised and weighed. All animal experiments were carried out according to the Austrian guidelines for animal care and protection.

*Other methods*

Immunoblotting analysis, immunofluorescence staining, lysosomal enzyme secretion studies, subcellular fractionation, soft agar colony formation and invasion assays were performed essentially as described [9,11-14]. Statistical analyses were performed using Student's *t*-test, with *** *p* < 0.001, ** *p* < 0.01 and * *p* < 0.05 being considered significant.

*References*

[1] Yeoh GC et al. Gene expression in clonally derived cell lines produced by *in vitro* transformation of rat fetal hepatocytes: isolation of cell lines which retain liver-specific markers. Cancer Res 1990;50:7593-7602.

[2] Tee LB, Smith PG, Yeoh GC. Expression of alpha, mu and pi class glutathione S-transferase in oval and ductal cells in liver of rats placed on a choline-deficient, ethionine-supplemented diet. Carcinogenesis 1992;13:1879-1885.

[3] Dumble ML et al. Generation and characterization of p53 null transformed hepatic progenitor cells: oval cells give rise to hepatocellular carcinoma. Carcinogenesis 2002;23:435-445.

[4] Hilliard CM, Fletcher S, Yeoh GC. Calcium phosphate transfection and cell-specific expression of heterologous genes in primary fetal rat hepatocytes. Int J Biochem Cell Biol 1996;28:639-650.

[5] Tan SS. Liver-specific and position-effect expression of a retinol-binding protein-lacZ fusion gene (RBP-lacZ) in transgenic mice. Dev Biol 1991;146:24-37.

[6] Mikula M et al. Immortalized p19ARF null hepatocytes restore liver injury and generate hepatic progenitors after transplantation. Hepatology 2004;39:628-634.

[7] Probst OC et al. The 46-kDa mannose 6-phosphate receptor does not depend on endosomal acidification for delivery of hydrolases to lysosomes. J Cell Sci 2006;119:4935-4943.

[8] Ho SN et al. Site-directed mutagenesis by overlap extension using the polymerase chain reaction. Gene 1989;77:51-59.

[9] Probst OC et al. The mannose 6-phosphate/insulin-like growth factor II receptor restricts the tumourigenicity and invasiveness of squamous cell carcinoma cells. Int J Cancer 2009;124:2559-2567.

[10] Valster A et al. Cell migration and invasion assays. Methods 2005;37:208-215.

[11] Mach L et al. Proteolytic processing and glycosylation of cathepsin B. The role of the primary structure of the latent precursor and of the carbohydrate moiety for cell-type-specific molecular forms of the enzyme. Biochem J 1992;282:577-582.

[12] Mach L, Mort JS, Glössl J. Maturation of human procathepsin B. Proenzyme activation and proteolytic processing of the precursor to the mature proteinase, in vitro, are primarily unimolecular processes. J Biol Chem 1994;269:13030-13035.

[13] Schähs P et al. Cellular repressor of E1A-stimulated genes is a bona fide lysosomal protein which undergoes proteolytic maturation during biosynthesis. Exp Cell Res 2008;314:3036-3047.

[14] Schmid JA et al. Accumulation of sialic acid in endocytic compartments interferes with the formation of mature lysosomes. Impaired proteolytic processing of cathepsin B in fibroblasts of patients with lysosomal sialic acid storage disease. J Biol Chem 1999;274:19063-19071.
