# Supplementary material for: M6P/IGF2R modulates the invasiveness of liver cells via its capacity to bind mannose 6-phosphate residues
Source: J Hepatol. 2012 Aug;57(2):337–43. doi: 10.1016/j.jhep.2012.03.026 (PMC3401376; doi:10.1016/j.jhep.2012.03.026)
Supplement: Supplementary Table 1 — M6P/IGF2R content of FRL14 cells expressing human M6P/IGF2R. [file mmc2.doc]

**Supplementary Table 1**. **M6P/IGF2R content of FRL14 cells expressing human M6P/IGF2R.**

| Cell line | M6P/IGF2R content [pmol/mg] |
| --- | --- |
| FRL14/IGF2R wt-1 | 2.5 |
| FRL14/IGF2R wt-2 | 2.1 |
| FRL14/IGF2R dom11^mut^ | 3.5 |
| FRL14/IGF2R dom3/9^mut^-1 | 4.7 |
| FRL14/IGF2R dom3/9^mut^-2 | 2.3 |
