# Supplementary material for: M6P/IGF2R modulates the invasiveness of liver cells via its capacity to bind mannose 6-phosphate residues
Source: J Hepatol. 2012 Aug;57(2):337–43. doi: 10.1016/j.jhep.2012.03.026 (PMC3401376; doi:10.1016/j.jhep.2012.03.026)
Supplement: Supplementary Table 2 — PCR primer sets for generation of mutant M6P/IGF2R cDNAs. [file mmc3.doc]

**Supplementary Table 2**. **PCR primer sets for generation of mutant M6P/IGF2R cDNAs.**

| Dom3^mut^: |  |
| --- | --- |
| M6P/IGF2R ^840-1434^ | 5’-CACTGCCGCCTGCCTGGTA-3’ (forward)  5’-GACGCTCATCTTCTGAAACCCTG-3’ (reverse) |
| M6P/IGF2R ^1411-2709^ | 5’-TCAGGGTTTCAGAAGATGAGCGTC-3’ (forward)  5’-TCAGTAACTTGGGAATGGCAAAG-3’ (reverse) |
| M6P/IGF2R ^840-2709^ | 5’-CACTGCCGCCTGCCTGGTA-3’ (forward)  5’-TCAGTAACTTGGGAATGGCAAAG-3’ (reverse) |
| Dom9^mut^: |  |
| M6P/IGF2R ^2524-4137^ | 5’-AACTGGTATGCCATGGACAACTCAGGGGAA-3’(forward)  5’-GAAGATGGCTGTGGATTTCTGATAAACCTTATGGCAAG  TGTCCC-3’ (reverse) |
| M6P/IGF2R ^4094-5063^ | 5’-GGGACACTTGCCATAAGGTTTATCAGAAATCCACAGCCA  TCTTC-3’ (forward)  5’-GAGAAGAAGAGAGTGCATGTCTGCTTGTCCA-3’ (reverse) |
| M6P/IGF2R ^2524-5063^ | 5’-AACTGGTATGCCATGGACAACTCAGGGGAA-3’ (forward)  5’-GAGAAGAAGAGAGTGCATGTCTGCTTGTCCA-3’ (reverse) |
| Dom11^mut^: |  |
| M6P/IGF2R ^4848-4876^ | 5’-TGGACAGACCAGGACTAGCGTGGGCAAGG-3’ (forward)  5’-CCTTGCCCACGCTAGTCCTGGTCTGTCCA-3’ (reverse) |
